# Effect of 910-MHz Electromagnetic Field on Rat Bone Marrow

**DOI:** 10.1100/tsw.2004.178

**Published:** 2004-10-20

**Authors:** George Demsia, Dimitris Vlastos, Demetrios P. Matthopoulos

**Affiliations:** Department of Environmental and Natural Resources Management, Ioannina University, Seferi 2, Agrinio 30100, Greece

**Keywords:** bone marrow, micronuclei, PCEs, nonionizing radiation, EMF, polymorphonuclear cells, rats

## Abstract

Aiming to investigate the possibility of electromagnetic fields (EMF) developed by nonionizing radiation to be a noxious agent capable of inducing genotoxicity to humans, in the current study we have investigated the effect of 910-MHz EMF in rat bone marrow. Rats were exposed daily for 2 h over a period of 30 consecutive days. Studying bone marrow smears from EMF-exposed and sham-exposed animals, we observed an almost threefold increase of micronuclei (MN) in polychromatic erythrocytes (PCEs) after EMF exposure. An induction of MN was also observed in polymorphonuclear cells. The induction of MN in female rats was less than that in male rats. The results indicate that 910-MHz EMF could be considered as a noxious agent capable of producing genotoxic effects.

## INTRODUCTION

The increased urbanization of the last 2 centuries in relation to human dependence to a very large variety of electrical appliances has created an increased concern about the possible side effects of the exposure to electromagnetic fields (EMF)[1]. Over the years, many studies have been performed to evaluate the possible EMF side effects to humans and other living beings. Thus, risk-assessment guidelines were set up, taking into account that EMF are distinguished into ionizing and nonionizing radiations[2,3,4]. The development of new appliances that produce EMF around them urged scientists to re-evaluate the instituted risk-assessment guidelines after extensive studies on side effects.

During the last 2 decades, a new gadget — the mobile phone — has been introduced into our daily lives. The dramatic increase in mobile phone users during the last 5 years has raised increased concern over the possible side effects of nonionizing radiation. Society's concern became more prominent as the number of younger users has overcome those of middle and older ages.

Mobile phones are in service in the frequency spectrum of 900–1800 MHz in the Time Division Multiple Access (TDMA) technique applied in Europe. In this frequency spectrum, there are a limited number of studies regarding the major side effects, i.e., thermal and nonthermal. Reports in the past under careful consideration proved to deal with thermal effects, although initially this was not realized[5,6,7,8]. However, little has been done on the nonthermal side effects[9,10].

The majority of the published work on nonionizing EMF exposure deals with frequencies used either by normal electrical appliances or by microwave ovens. Published reports are contradictory. The strategies applied either experimental animals or cell cultures and blood samples. Positive and negative side effects for the same frequencies were reported that were dependent on the animals and tissues used, as well as the techniques for the estimation of the side effects[2,11]. Due to these contradictory reports, further studies should be carried out.

A system commonly applied for *in vivo* evaluation of the genotoxicity of various chemicals or other noxious conditions is the rodent micronucleus test. A restricted number of micronuclei (MN) appear under normal conditions in various tissues and cells that increase when noxious agents affect a living system[12,13]. The development of MN is a result of either clastogenic (i.e., MN arising from chromosomal fragments) or aneugenic (i.e., MN occurring from whole chromosomes that are not correctly integrated into the daughter nuclei during mitosis) side effects. As an *in vivo* genotoxicity test system, it has been used not only in peripheral blood, but in bone marrow cells as well[14].

In the frequency range of 900–1800 MHz, there are very few reported results that cannot give a clear-cut answer and confirm whether or not there is a positive mutagenic or genotoxic effect[2,11]. However, in the 1999 annual meeting of the Bioelectromagnetics Society in Long Beach, California, a significant increase in the incidence of MN in human peripheral blood lymphocytes after continuous *in vitro* exposure for 24 h to radiofrequency radiation at frequencies used for cellular telephone communications was reported[15,16].

In the present study, irradiated rat bone marrow cells were analyzed with two objectives in mind. First, to study whether mobile phone frequency EMF can be considered as a noxious agent. Second, to supply additional information in the present state on the possible genotoxic effect EMF developed by mobile phones. We arranged our system in a way that thermal side effects were omitted so that our observations would be focused on the nonthermal side effects.

## MATERIALS AND METHODS

One-year-old male and female Wistar rats were used. Animals were housed in groups of four in Plexiglas cages kept in the animal house of Ioannina University Medical School under well-controlled conditions of temperature at 20 $\pm$ 1°C, 50% relative humidity, and 12-h light and dark cycles.

Animals were exposed for 2 h/day for 30 consecutive days to a continuous 910-MHz EMF in specially designed Plexiglas cages. Animals were restricted from free movements in order to be equally exposed to the EMF energy. The cages were placed at a distance of 5 mm from a $\lambda/2$ emitting antenna. During the experimental procedure, the maximum SAR value using the Finite Difference Time Domain (FDTD) analysis[17] was calculated and found to be 0.42 W/Kg (10 g) of body weight. Care was taken so that the rat tails did not interfere with the experimental conditions. Four cages were arranged next to the antenna in such a way that the animals were exposed to the maximum of EMF energy at the area of the head, while the hind part of the animal body received approximately 15 db less than the head[17].

Sham-exposed animals were kept in the same type of cages for the same period every day. Animals were starved from food and water during exposure. Throughout the experiments, care was taken to minimize pain or discomfort. All studies were approved by the Ioannina University Institutional Animal Care and Use Committee.

One day after the last exposure, the animals were sacrificed after ethyl ether anesthesia. Both femurs were isolated, cleared from the attached tissues, and the bone marrow was collected after cutting the epiphyseal cartilage with mild centrifugation in 5-ml centrifuge tubes containing 2 ml of fetal calf serum. Pelleted marrow cells were resuspended in 4-ml fetal calf serum and centrifuged at 1000 rpm for 3 min.

Cells resuspended in a small amount of serum were spread onto microscope slides, air-dried, methanol fixed, and stained with May-Grunwald followed by Giemsa[18].

Microscopic observations were carried out on a Leica microscope with Plan objective at 1000× magnification. At least 3.000 erythrocytes were scored per slide for the presence of MN in polychromatic erythrocytes (PCEs). For each group, three slides were examined per animal to a total of at least $9 \times 10^3$ erythrocytes. Photographs were taken with a 4× additional magnification applied by the camera.

In parallel, fixed slides were stained with an aqueous solution of 1 mg/ml acridine orange, covered with cover slips, and examined by fluorescent microscopy.

The one-way ANOVA test of the Origin 7.0 software (OriginLab Corporation, Northampton, USA) was applied for the statistical analysis between the studied groups.

## RESULTS

The frequency of PCEs over the mature erythrocytes was calculated in control and experimental animals in order to evaluate any direct effect of irradiation in rat erythopoiesis. The resulted percentage of PCEs in the irradiated animals was found to be within the range of the control animals.

Table 1 depicts the number of MN observed in bone marrow spreads from control and irradiated animals. Fig. 1 shows micronucleated PCEs from irradiated rats (a) and micronucleated polymorphonuclear cells (b). Analyzing several marrow spreads per irradiated animals, the main observation was that apart from the micronucleated PCEs, a large number contained amorphous inclusions that were omitted from our calculations. Only rounded and well-defined inclusions stained with the May-Grunwald / Giemsa double stain were assigned as MN in PCEs (Fig. 1a) and these were the ones stained with the acridine orange fluorescent stain.

**Table 1.** Micronuclei calculated per ‰ in control and irradiated rats.

| Animals | RBCs X $10^3$ | ‰ MN ± sd |
|---|---|---|
| Control ♂ | 36 | 3.27 ± 0.68 |
| Control ♀ | 36 | 2.18 ± 0.14 |
| Irradiated ♂ | 36 | 9.64 ± 0.32 |
| Irradiated ♀ | 36 | 5.56 ± 0.51 |
| RBCs: Red Blood Cells, MN: micronuclei, sd: standard deviation | | |

The statistical analysis of the induced MN per 1000 PCEs is shown in Table 2.

Our data revealed an induction of micronucleated PCEs in bone marrow from irradiated rats. An important observation comes from the fact that there is a significant difference between irradiated male and female rats ($p < 0.001$), whereas a corresponding difference was not observed in the sham-exposed animals ($p = 0.190$).

Systematic observation of bone marrow smears from irradiated animals revealed an induction of MN in polymorphonuclear cells (Fig. 1b). Micronucleated polymorphonuclear cells were not detected in the sham-exposed animals. The distinction of MN in these cells is difficult as they are usually confused with normal nuclear lobes or projections, thus they have not been presently calculated.

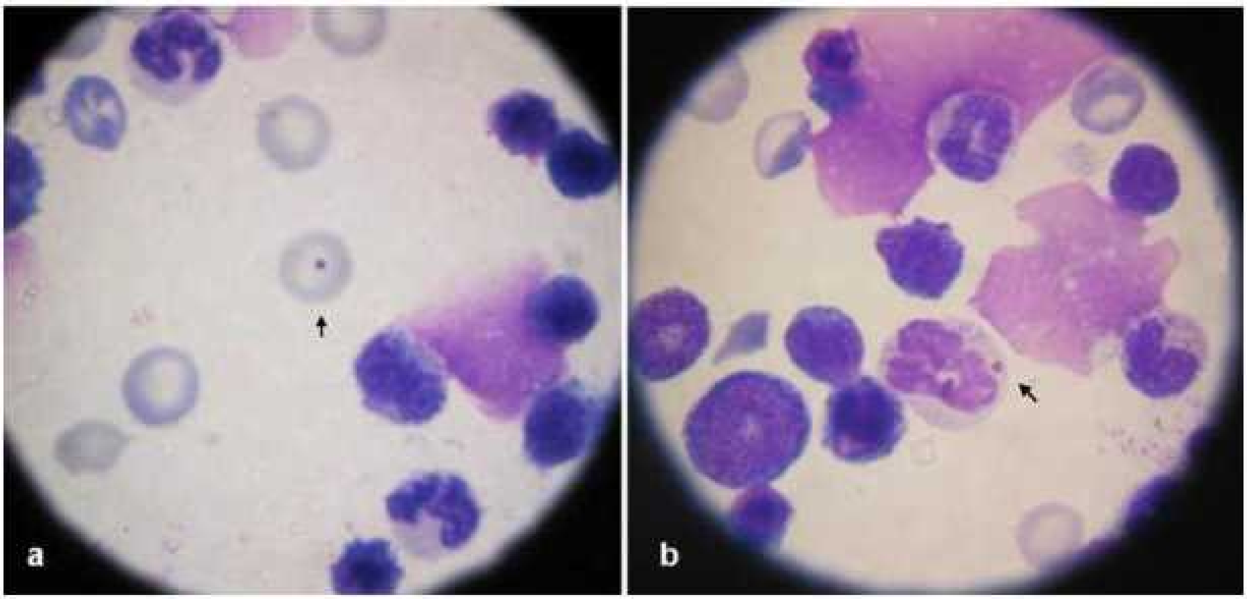

**FIGURE 1.** Micronucleated bone marrow cells. (a) Micronucleus in a PCE, (b) micronucleus in a polymorphonuclear cell. Arrows indicate the micronucleated cells.

**Table 2.** Analysis of variance of the induced micronuclei in polychromatic erythrocytes in control and irradiated rats.

| Animals | F | p |
|---|---|---|
| Control ♂ / ♀ | 2.49 | 0.190 |
| Irradiated ♂ / ♀ | 46.16 | < 0.001 |
| ♂ Control / Irradiated | 86.67 | < 0.001 |
| ♀ Control / Irradiated | 30.58 | < 0.01 |

## DISCUSSION

During the last 10 years, serious concern has been raised over possible side effects of the nonionizing radiation frequency spectrum used for telecommunications. The increased popularity of mobile phones, especially among the younger population of the developed countries, raises discussions over health safety. Investigations carried out in the past could not reach clear-cut positive or negative results.

In our study, rats during irradiation were kept almost immobile not only due to the restricted space available to them but, in addition, due to the fact that their tails were taped against the base of their cages. Under the exposure conditions applied, the animals received a reduced energy dose along their bodies by almost 15 db as evaluated by the FDTD analysis. The experimental conditions applied[17] were designed so that the body temperature of the animals was not affected by the EMF and thus the observed alterations were the result of EMF nonthermal side effects.

Studying the effect of 910-MHz radiation on rat bone marrow, we did not observe any alteration on the production rate of erythrocyte precursors. The proportion of PCEs and mature erythrocytes in the irradiated animals did not alter compared to controls. It has been reported that there was no effect to the erythrocytopoiesis in mice even after 18 months exposure to 2450-MHz radiofrequency[19]. However, in rats, in a shorter exposure period, an increased influx of immature erythrocytes, PCEs, into the peripheral circulation was observed over a period of 2 weeks that finally balanced to the physiological range by the fourth week of irradiation[20]. An adaptive or recovery mechanism has been proposed to be associated with subchronic radiation exposure to 2450-MHz radiofrequency.

Within the framework of our experiments, in irradiated male rats, an almost threefold induction of MN in bone marrow immature erythrocytes was observed, while in females, the induction of MN was almost two and a half times higher compared to control animals. MN, being the result of noxious agents causing clastogenic or aneugenic side effects[12,13], appear in precursor red blood cells between 10 and 24 h post-treatment. It has to be proved that EMF developed by mobile phones belong to noxious agents with or without additive effects.

The statistical analysis indicated a considerable increase of MN in male rats, but not to the same extent in females. However, in both male and female, the induction was greater than in the sham-exposed animals. In parallel experiments, the overall brain collagen fibril architecture was disturbed in male rats on EMF exposure, but not in females (Tzaphlidou personal communication). Whether the observed EMF side effects are sex dependent or not requires further investigation. Such differences have been reported for mice in genotoxicity studies[21]. The response of the various strains of mice to the spontaneous induction of MN was found to vary. Thus, at least using mice, care has to be taken on the mouse strain used in experiments. In the mean time, male mice were proposed to be more sensitive for micronucleus induction studies and it was suggested that they should be used exclusively for the assay in most cases[22,23]. However, although differences in the response to several noxious compounds were observed, it was finally proposed that equal numbers of male and female mice should be included in genotoxicity studies[21].

Nonionizing radiation in the area that microwave ovens are performing produced contradicting results. 2450 MHz at a whole body nominal SAR of 12 W/Kg for 24 h, applying a different irradiation schedule, did not induce MN in either rat bone marrow or peripheral blood cells[24]. However, MN induction was observed in peripheral blood of rats exposed to 2450 MHz, at a whole body nominal SAR of 1–2 W/Kg, radiofrequency radiation for 2 h/day over a period of 8 consecutive days although on prolonged exposure up to 30 days, the observed induction disappeared[20]. It should be noted that, in rats like in humans, abnormally developed red blood cells appearing in the circulation are eliminated by the spleen[25]. Exposing human lymphocytes *in vitro* to microwave radiation (2.45 and 7.7 GHz at a power density of 30 mW/cm$^2$ for 30 or 60 min) resulted in an increase in MN frequency compared to control cultures[26]. However, exposing the same cell system *in vitro* at low frequency microwaves (380, 900, and 1800 MHz) modulated in amplitude at 0.73–3.1 MHz did not produce any variation in the spontaneous level of sister chromatid exchanges[27].

Extremely low-frequency magnetic fields (ELM-MF), although they do not induce MN in studied systems[28], should be considered as enhancers for tumor promoters or initiators[29,30,31].

In our study, we observed a rather high number of bone marrow polymorphonuclear cells bearing MN in the irradiated rats. After careful observation, MN in these cells could be distinguished from normal nuclear lobes or projections. Restricting the analysis only to nucleated bone marrow cells is rather unreliable if the effects of chromosome breaking agents are small[18]. The appearance of MN in bone marrow polymorphonuclear cells rather than PCEs has not been previously reported. Further studies are in progress in order to clarify the induction of MN in nucleated bone marrow cells.

Investigations in order to verify whether or not there are adverse side effects to living organisms from exposure to the range of nonionizing radiation between 800 and 1800 MHz are scattered. However, contradictory reports stress that more research is needed[2,11].

In conclusion, under the 910-MHz frequency radiation exposure conditions used in the present study, the results indicate an approximately threefold increase of MN in bone marrow PCEs in exposed rats compared to sham-exposed animals. An induction of MN in polymorphonuclear cells is also apparent in the bone marrow. Our results support the possibility that mobile phone frequency EMF is a noxious agent capable of producing genotoxic results in living systems.

## ACKNOWLEDGMENTS

Thanks are due to Mr. E. Fotiou for helping with animals' irradiation and to Prof. M. Tzaphlidou for valuable comments on the manuscript.
